# The Transmembrane Serine Protease HAT-like 4 Is Important for Epidermal Barrier Function to Prevent Body Fluid Loss

**DOI:** 10.1038/srep45262

**Published:** 2017-03-24

**Authors:** Zhiwei Zhang, Yae Hu, Ruhong Yan, Liang Dong, Yizhi Jiang, Zhichao Zhou, Meng Liu, Tiantian Zhou, Ningzheng Dong, Qingyu Wu

**Affiliations:** 1 Cyrus Tang Hematology Center, MOE Engineering Center of Hematological Disease, Collaborative Innovation Center of Hematology, Soochow University, Suzhou, China; 2 MOH Key Lab of Thrombosis and Hemostasis, Jiangsu Institute of Hematology, the First Affiliated Hospital of Soochow University, Suzhou, China; 3 Molecular Cardiology, Cleveland Clinic, Cleveland, Ohio, USA.

## Abstract

Membrane-bound proteases are essential for epidermal integrity. Human airway trypsin-like protease 4 (HAT-L4) is a type II transmembrane serine protease. Currently, its biochemical property, cellular distribution and physiological function remain unknown. Here we examined HAT-L4 expression and function *in vitro* and *in vivo*. In Western analysis, HAT-L4 expressed in transfected CHO cells appeared as a 48-kDa protein. Flow cytometry confirmed HAT-L4 expression on the cell surface with the expected membrane topology. RT-PCR and immunostaining experiments indicated that HAT-L4 was expressed in epithelial cells and exocrine glands in tissues including skin, esophagus, trachea, tongue, eye, bladder, testis and uterus. In the skin, HAT-L4 expression was abundant in keratinocytes and sebaceous glands. We generated HAT-L4 knockout mice by disrupting the *Tmprss11f* gene encoding HAT-L4. HAT-L4 knockout mice were viable and fertile. No defects were found in HAT-L4 knockout mice in hair growth, wound healing, water repulsion and body temperature regulation. Compared with wild-type controls, HAT-L4-deficient newborn mice had greater body fluid loss and higher mortality in a trans-epidermal body fluid loss test. In metabolic studies, HAT-L4-deficient adult mice drank water more frequently than wild-type controls did. These results indicate that HAT-L4 is important in epidermal barrier function to prevent body fluid loss.

Proteolysis is essential in biology. More than 2% of genes in the human genome encode proteolytic enzymes^1^, among which trypsin-like serine proteases represent a major superfamily. It is well established that trypsin-like proteases are important for many physiological processes such as food digestion, blood coagulation, wound healing, and inflammatory responses^2^,^3^. Recently, a subgroup of type II transmembrane serine proteases (TTSPs) have been identified within the trypsin superfamily^4^,^5^. All TTSPs consist of an N-terminal cytoplasmic tail, a single-span transmembrane domain and an extracellular region that contains various protein modules and a C-terminal serine protease domain. Unlike trypsin, which is a secreted protein, TTSPs are anchored on the cell surface via their transmembrane domains.

In humans, there are 17 TTSPs identified to date^4^. Based on their extracellular protein domain arrangements, human TTSPs can be divided into four subgroups, *i.e*. human airway trypsin (HAT)-like protease/differentially expressed in squamous cell carcinoma (DESC), hepsin/TMPRSS (transmembrane protease/serine), matriptase, and corin subgroups^4^. Many of these TTSPs have been reported to play important roles in health and disease^6^,^7^,^8^,^9^,^10^,^11^. For example, matriptase is critical for epithelial integrity and function^12^. Matriptase-2 is indispensable for iron metabolism^13^. Corin is essential for sodium homeostasis and normal blood pressure^14^,^15^. Hepsin and TMPRSS3 are important for normal hearing^16^,^17^. Defects in these TTSPs may lead to major diseases such as skin disorders^18^,^19^, congenital or inflammatory diarrheal diseases^20^,^21^,^22^, iron-deficiency anaemia^23^,^24^,^25^, hypertension^26^,^27^,^28^,^29^, and hearing loss^16^.

The HAT/DESC subgroup of human TTSPs has five members, *i.e*. HAT, DESC, TMPRSS11A, HAT-like 4 (HAT-L4) and HAT-like 5, all of which share a similar overall domain arrangement: a cytoplasmic tail of 18–28 amino acids, a transmembrane domain and an extracellular stem region containing a SEA (sea urchin sperm protein/enteropeptidase/agrin) domain and a C-terminal serine protease domain^4^. The genes encoding these proteases reside in tandem at human chromosome 4q13.2, suggesting that these genes may be evolved from gene duplication. To date, the functional significance of this subgroup of TTSPs remains poorly understood. HAT, for example, was identified in human airway epithelial tissues^30^. In biochemical and cellular studies, HAT was shown to cleave different substrates such as protease activated receptor-2, the urokinase receptor and pro-gamma-melanotropin^31^,^32^,^33^. In mice, however, disrupting the *Tmprss11d* gene encoding HAT did not reveal any detectable abnormalities in embryonic development and post-natal survival^34^. It is unknown if the TTSPs of the HAT/DESC subgroup are redundant or their functions are required only upon specific environmental challenges.

HAT-L4 is a TTSP of the HAT/DESC subgroup^4^. In PCR studies, HAT-L4 mRNA was found in tissues including skin, esophagus, tongue, testis, stomach and bladder^34^. The biological function of HAT-L4 has yet to be defined. In this study, we expressed and analyzed recombinant human HAT-L4 by Western blotting and flow cytometry. We also did immunohistochemistry (IHC) to examine cellular distribution of HAT-L4 expression in human tissues. Moreover, we generated and characterized the knockout (KO) mice, in which the *Tmprss11f* gene encoding HAT-L4 was disrupted by CRISPR/Cas9-based techniques. Our data show that HAT-L4 is expressed in epithelial cells and exocrine glands in multiple tissues including the skin and that HAT-L4 is important for epidermal barrier function to prevent body fluid loss in newborn mice.

## Results

### Analysis of Recombinant Human HAT-L4 Protein

**Figure 1:**
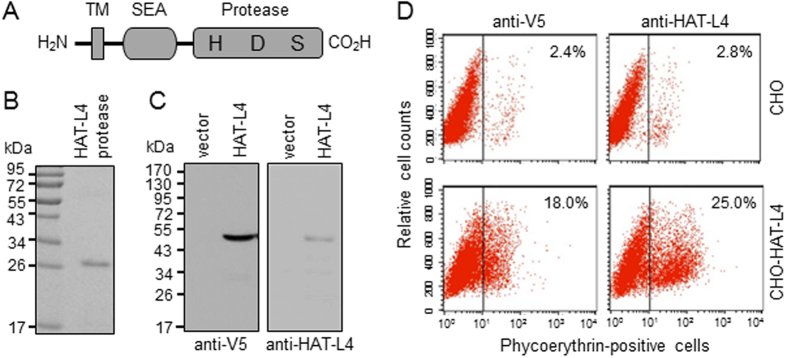
Analysis of Recombinant Human HAT-L4. (**A**) HAT-L4 protein domains. The transmembrane (TM), SEA and protease domains and active sites His (H), Asp (D) and Ser (S) are indicated. (**B**) A purified HAT-L4 fragment, consisting of the protease domain, was analyzed by SDS-PAGE and Coomassie blue staining. (**C**) A full-length recombinant human HAT-L4 containing a C-terminal V5 tag was expressed in CHO cells and analyzed by Western blotting using anti-V5 (*left panel*) and anti-HAT-L4 (*right panel*) antibodies. Vector-transfected CHO cells were used as controls. (**D**) Flow cytometric analysis of HAT-L4-expressing (*lower panels*) and vector-transfected (*upper panels*) CHO cells using anti-V5 (*left panels*) and anti-HAT-L4 (*right panels*) antibodies.

Human HAT-L4 is a polypeptide of 438 amino acids^4^. Figure 1A shows the predicted protein domain structure of HAT-L4. We expressed a human HAT-L4 fragment, consisting of the protease domain, in *Escherichia coli* and purified it with affinity chromatography. In SDS-PAGE analysis, the purified HAT-L4 fragment migrated as a band of ~28 kDa (Fig. 1B). The HAT-L4 fragment was used as an immunogen to make anti-HAT-L4 monoclonal antibodies. We next expressed the full-length human HAT-L4 in Chinese hamster ovary (CHO) cells. The recombinant HAT-L4 contained a C-terminal V5 tag. In Western blotting analysis, both anti-V5 and anti-HAT-L4 antibodies recognized a dominant band of ~48 kDa in lysates from the transfected CHO cells expressing the full-length HAT-L4 protein (Fig. 1C). Such a band was not detected in control lysates from vector-transfected CHO cells. In flow cytometric analysis, both the anti-V5 and anti-HAT-L4 antibodies detected recombinant human HAT-L4 on the transfected CHO cell surface (Fig. 1D, *lower panels*). The results are consistent with the predicted cell membrane topology of HAT-L4 with its C-terminus located extracellularly.

### HAT-L4 Expression in Human and Mouse Tissues

**Figure 2:**
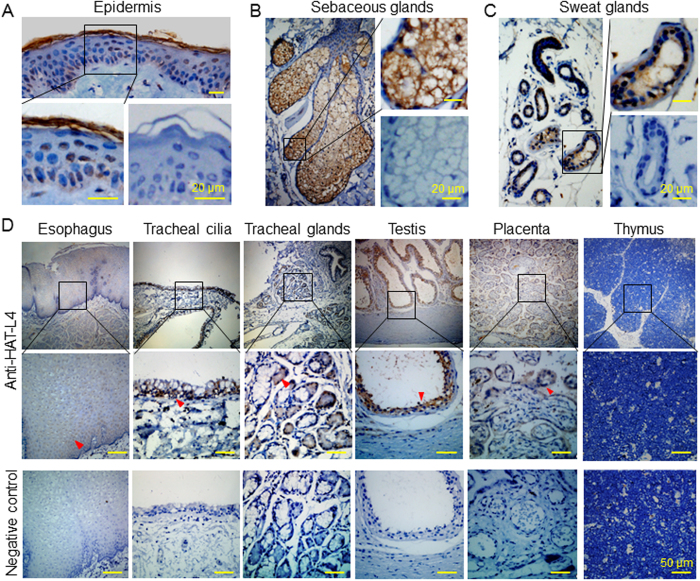
HAT-L4 protein expression in human tissues. Tissue sections were analyzed by IHC using an anti-HAT-L4 antibody. (**A**–**C**), HAT-L4 expression in skin tissues (**A**, epidermis; (**B**), sebaceous glands; (**C**), sweat glands) is indicated by brown staining. Negative controls without the primary antibody are shown in the right bottom panels. (**D**) HAT-L4 expression in esophagus, trachea, testis and placenta is indicated by brown staining (red arrowheads). No HAT-L4 staining was detected in thymus sections. Negative controls without the primary antibody are shown in panels of the bottom row. Scale bars: 20 μm in (**A**–**C**) and 50 μm in D.

**Figure 3:**
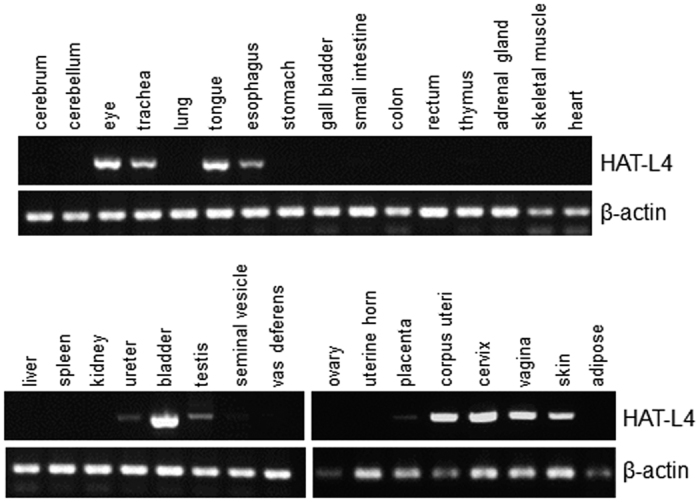
RT-PCR analysis of HAT-L4 mRNA expression in mouse tissues. Total RNAs were isolated from tissues of WT mice. HAT-L4 mRNA expression was analyzed by RT-PCR. Beta-actin mRNA expression was used as a positive control.

Previously, HAT-L4 mRNA expression was detected by RT-PCR in human and mouse tissues, including skin, tongue, esophagus, testis, placenta and cervix^34^. To understand cellular distributions of HAT-L4, we examined HAT-L4 protein in selected human tissues by IHC (Fig. 2). In skin sections, positive HAT-L4 staining was observed in the stratum corneum and epidermal keratinocytes (Fig. 2A) and in sebaceous (Fig. 2B) and sweat (Fig. 2C) glands. In addition, HAT-L4 staining was detected in esophageal squamous epithelial cells, tracheal cilia and submucosal glands, seminiferous epithelium, and placental chorionic villi (Fig. 2D). In contrast, HAT-L4 was not detected in the thymus (Fig. 2D). These data indicate that HAT-L4 is expressed in epithelial cells and exocrine glands in different human tissues. We next did an RT-PCR survey of HAL-L4 expression in 32 mouse tissues. HAL-L4 mRNA was detected in multiple tissues including eye, trachea, tongue, esophagus, ureter, bladder, testis, placenta, uterus, vagina and skin (Fig. 3).

### Generation of *Tmpress11f* KO Mice

**Figure 4:**
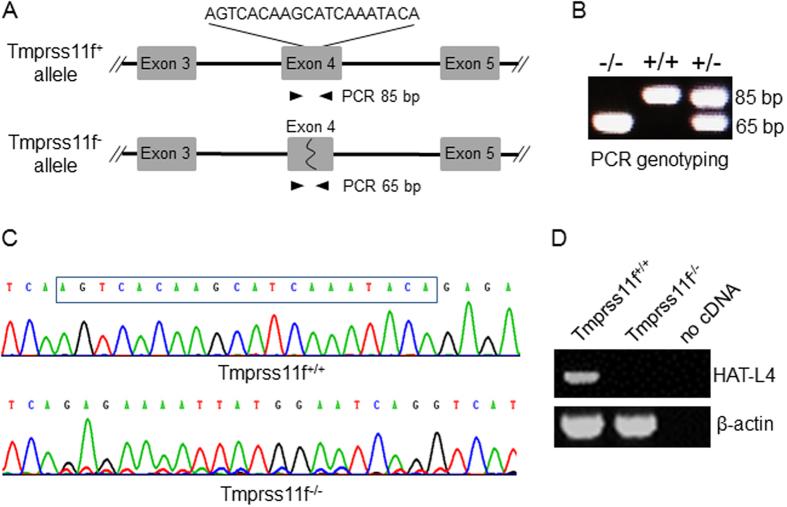
Disruption of the mouse *Tmprss11f* gene. (**A**) Illustration of CRISPR/Cas9-based targeting strategy to delete a 20-bp sequence in *Tmprss11f* exon 4. The WT (*Tmprss11f*^+^) allele (*upper*), the targeted (*Tmprss11f*^−^) allele (*lower*) and PCR primers (*black arrowheads*) for genotyping are indicated. (**B**) Representative PCR genotyping results of *Tmprss11f*^+/+^, *Tmprss11f*^+/−^ and *Tmprss11f*^−/−^ mice. PCR fragments were analyzed in ethidium bromide-stained agarose gel. (**C**) DNA sequencing traces confirming *Tmprss11f*^−^ (*upper*) and *Tmprss11f*^−/−^ (*lower*) alleles. The targeted nucleotide sequence in the *Tmprss11f*^+/+^ allele (*upper, boxed*) was deleted in the *Tmprss11f*^−/−^ allele (*lower*). (**D**) RT-PCR analysis of HAT-L4 mRNA expression in tongues from *Tmprss11f*^+/+^ and *Tmprss11f*^−/−^ mice. A negative control without cDNA templates in RT-PCR (no cDNA) was included in the experiment. Beta-actin mRNA expression was used as a positive control.

The mouse *Tmprss11f* gene consists of 11 exons. To generate *Tmprss11f* KO mice, a CRISPR/Cas9-based targeting RNA was designed to delete a 20 bp-nucleotide sequence in *Tmprss11f* exon 4 (Fig. 4A), which encodes amino acids 68–73 (Val-Thr-Ser-Ile-Lys-Tyr) in the extracellular SEA domain of mouse HAT-L4. The deletion is expected to shift the downstream protein coding sequence, thereby resulting in a null *Tmprss11f*^−/−^ allele. The guide and targeting RNAs were injected into C57BL/6 fertilized oocytes to generate *Tmprss11f*^+/−^ mice, which were bred to produce *Tmprss11f*^+/+^, *Tmprss11f*^+/−^ and *Tmprss11f*^−/−^ mice, as indicated by PCR genotyping with oligonucleotide primers flanking the exon 4 deletion site (Fig. 4A and B). The PCR fragments representing the *Tmprss11f*^+/+^ and *Tmprss11f*^−/−^ alleles were verified by DNA sequencing to confirm the intended deletion (Fig. 4C). To examine HAT-L4 mRNA expression in WT and *Tmprss11f* KO mice, total RNAs were isolated from tongues, one of the tissues in which HAT-L4 mRNA expression was abundant (Fig. 3). RT-PCR detected HAT-L4 mRNA in *Tmprss11f*^+/+^ mice but not *Tmprss11f*^−/−^ mice (Fig. 4D).

### Embryonic Development, Post-natal Growth, Fertility and Long-term Survival of *Tmprss11f* KO Mice

**Figure 5:**
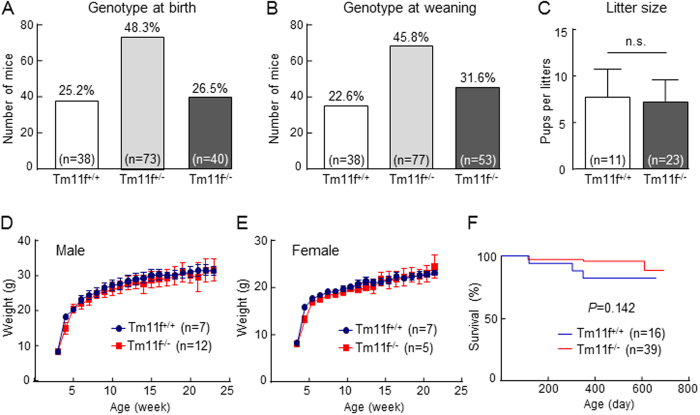
Embryonic development, fertility, post-natal growth and survival of *Tmprss11f* KO mice. (**A**,**B**) *Tmprss11f*^+/+^ (Tm11f^+/+^), *Tmprss11f*^+/−^ (Tm11f^+/−^) and *Tmprss11f*^−/−^ (Tm11f^−/−^) genotype distribution among offspring from *Tmprss11f*^+/−^ mating, as analyzed at birth (**A**) and weaning (**B**). (**C**) Litter sizes of *Tmprss11f*^+/+^ and *Tmprss11f*^−/−^ female mice mated with males of the same genotype. n.s., not significant. (**D**,**E**) Body weight gains in male (**D**) and female (**E**) *Tmprss11f*^+/+^ and *Tmprss11f*^−/−^ mice. (**F**) Long-term survival rates in *Tmprss11f*^+/+^ and *Tmprss11f*^−/−^ mice. The number of mice in each study group is indicated. In D-F, the differences between *Tmprss11f*^+/+^ and *Tmprss11f*^−/−^ mice were not statistically significant.

To examine the functional importance of HAT-L4, we bred *Tmprss11f*^+/−^ mice and genotyped offspring by PCR. Of 151 offspring from 18 litters genotyped at birth, 38 (25.2%) were *Tmprss11f*^+/+^, 73 (48.3%) were *Tmprss11f*^+/−^ and 40 (26.5%) were *Tmprss11f*^−/−^ (Fig. 5A), which was in line with the expected Mendelian ratio of inheritance. A similar Mendelian ratio was observed when the genotyping was done at the weaning (post-natal day 21). Of 168 offspring from 20 litters, 38 (22.6%) were *Tmprss11f*^+/+^, 77 (45.8%) were *Tmprss11f*^+/−^ and 53 (31.6%) were *Tmprss11f*^−/−^ (Fig. 5B). In mice of all three genotypes, male to female ratio was approximately 1:1. Both *Tmprss11f*^−/−^ males and females were fertile and produced viable offspring. We compared litter sizes from *Tmprss11f*^+/+^ and *Tmprss11f*^−/−^ females that were mated with males of the same genotype. Litter sizes were 7.6 ± 3.1 from *Tmprss11f*^+/+^ females (n = 11) and 7.2 ± 2.3 from *Tmprss11f*^−/−^ females (n = 23) (*P* = 0.7) (Fig. 5C). *Tmprss11f*^−/−^ male and female mice grew normally, as indicated by similar body weight gains to those in *Tmprss11f*^+/+^ male and female littermates (Fig. 5D and E). *Tmprss11f*^+/+^ and *Tmprss11f*^−/−^ mice also had similar long-term survival rates when monitored over 600 days (Fig. 5F). These results indicate that HAT-L4 is dispensable for embryonic development, post-natal growth, fertility and long-term survival in mice.

### Histological Examination and Blood Chemistry Analysis

**Figure 6:**
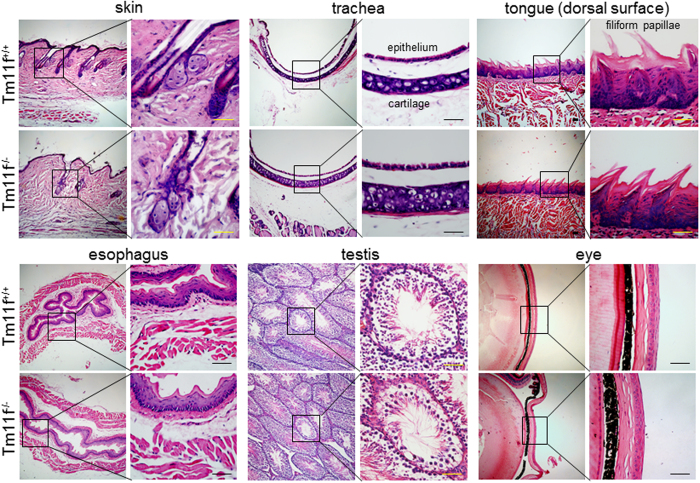
Histological analysis of *Tmprss11f* KO mouse tissues. Selected tissues from *Tmprss11f*^+/+^ (Tm11f^+/+^) and *Tmprss11f*^−/−^ (Tm11f^−/−^) mice were sectioned and stained with H&E. For each tissue, a high magnification picture is shown on the right side. Data are representative of the results from at least three individual mice per genotype. No significant morphological differences were observed between *Tmprss11f*^+/+^ and *Tmprss11f*^−/−^ mice in the tissues examined. Scale bars: 50 μm.

*Tmprss11f*^−/−^ mice did not exhibit apparent physical abnormalities. In 2–4-month-old *Tmprss11f*^−/−^ mice, necropsies did not find gross abnormalities in major organs such as heart, liver, lung, kidney, esophagus and spleen. Histological analysis was done in tissues where HAT-L4 expression was detected, including skin, trachea, tongue, esophagus, testis and eye. Tissue morphologies in hematoxylin and eosin (H&E)-stained sections from *Tmprss11f*^−/−^ mice were indistinguishable from those from *Tmprss11f*^+/+^ mice (Fig. 6). In blood chemistry analysis, values for total bilirubin, total protein, albumin, globulin, alanine aminotransferase, alkaline phosphatase, glucose and cholesterol were similar in gender-matched *Tmprss11f*^+/+^ and *Tmprss11f*^−/−^ mice (data not shown). In blood cell analysis, no significant differences between *Tmprss11f*^+/+^ and *Tmprss11f*^−/−^ mice were found in red blood cell, white blood cell, and platelet counts (data not shown).

### Analysis of Hair Growth, Wound Healing, Water Repulsion and Thermoregulation

**Figure 7:**
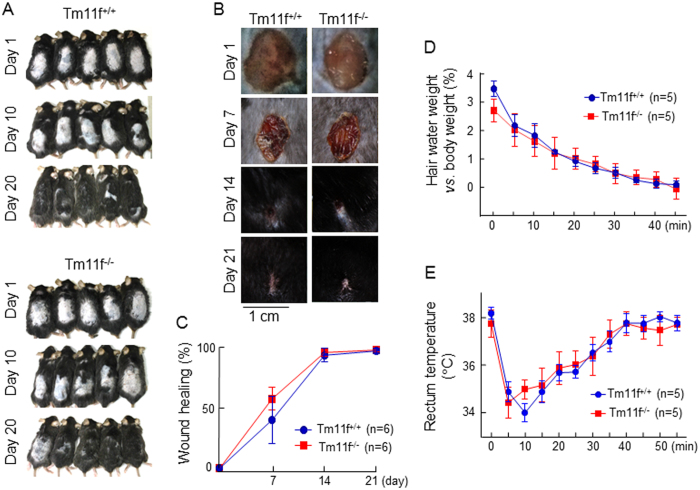
Hair growth, skin wound healing, hair water content and body temperature in Tmprss11f KO mice. (**A**) Hairs in dorsal areas were shaved in *Tmprss11f*^+/+^ (Tm11f^+/+^) and *Tmprss11f*^−/−^ (Tm11f^−/−^) mice. Hair growth at days 1, 10 and 20 was recorded photographically. (**B**) Dorsal skin wounds were created in *Tmprss11f*^+/+^ and *Tmprss11f*^−/−^ mice. Wound healing was recorded photographically over 21 days. The quantitative data of wound healing are shown in (**C**). The differences in wound healing rate between Tm11f^+/+^ and Tm11f^−/−^ mice were not statistically significant. (**D**,**E**) *Tmprss11f*^+/+^ and *Tmprss11f*^−/−^ mice were subjected to a water bath test. After 2 min in a water tank, the mice were taken out. Hair water weight (**D**) and body temperature (**E**) were measured over time. The differences in hair water evaporation and body temperature recovery between Tm11f^+/+^ and Tm11f^−/−^ mice were not statistically significant. The number of mice in each study group is indicated.

Membrane-bound serine proteases play important roles in skin integrity and function^12^,^35^. Like other members of the HAT/DESC subgroup, HAT-L4 is expressed in human and mouse skins (Figs 2 and 3). Histologically, no apparent differences were observed in H&E-stained sections between *Tmprss11f*^+/+^ and *Tmprss11f*^−/−^ mice (Fig. 6). To test the effect of HAT-L4-deficiency on skin functions, we analyzed hair growth in *Tmprss11f*^+/+^ and *Tmprss11f*^−/−^ mice. No significant difference in hair growth rates was found between the two groups of mice (Fig. 7A). Similar rates of wound healing were also observed in *Tmprss11f*^+/+^ and *Tmprss11f*^−/−^ mice (Fig. 7B and C). These results indicate that HAT-L4-deficiency does not impair hair growth and wound healing in mice.

We next examined the effect of HAT-L4-deficiency on water repulsion and thermoregulation. Adult *Tmprss11f*^+/+^ and *Tmprss11f*^−/−^ male mice were placed in a water tank at 30 °C. After 2 min, the mice were taken out, and hair drying rates and rectum temperature changes were monitored. Similar rates of hair drying and body temperature recovering were observed in *Tmprss11f*^+/+^ and *Tmprss11f*^−/−^ mice (Fig. 7D and E), indicating that HAT-L4 is not critical for water repulsion and body temperature regulation in adult mice under our experimental conditions.

### Trans-epidermal Fluid Loss and Water Drinking

**Figure 8:**
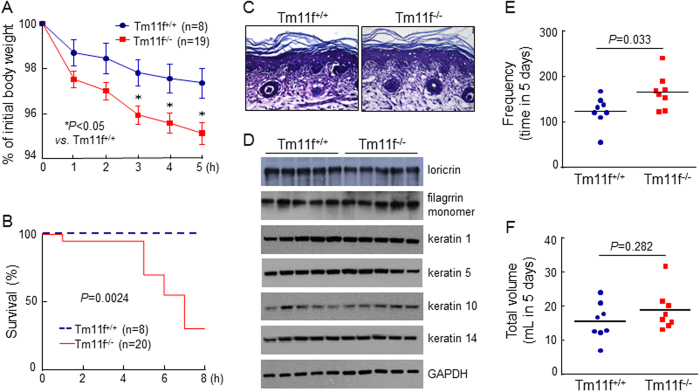
Trans-epidermal body fluid loss in newborn pups and water drinking in adult mice. (**A**) *Tmprss11f*^+/+^ (Tm11f^+/+^) and *Tmprss11f*^−/−^ (Tm11f^−/−^) newborn pups were placed in an incubator at 37 °C. Body weights were measured over time. The data are presented as % of initial body weight. *P < 0.05 *vs*. Tm11f^+/+^ mice at the same time point. (**B**) Survival rates of *Tmprss11f*^+/+^ and *Tmprss11f*^−/−^ newborn pups at 37 °C over time. (**C**) Histological analysis of epidermal structures in H&E-stained sections from *Tmprss11f*^+/+^ and *Tmprss11f*^−/−^ newborn mice. (**D**) Western blotting analysis of major epidermal proteins in *Tmprss11f*^+/+^ and *Tmprss11f*^−/−^ mice. (**E**,**F**) Adult *Tmprss11f*^+/+^ and *Tmprss11f*^−/−^ mice in metabolic cages were monitored for water-drinking frequencies (**E**) and total water intake volume (**F**) over 5 days. Each study group included 8 mice, as indicated by individual dots.

The skin serves as a barrier to prevent body fluid loss. This function is particularly important in newborn mice that are prone to dehydration. In a trans-epidermal fluid loss test performed at 37 °C, *Tmprss11f*^−/−^ newborn mice had more body fluid loss compared with that in *Tmprss11f*^+/+^ controls, as indicated by greater body weight decreases over a 5-h period (Fig. 8A). The dehydration caused >60% mortality in *Tmprss11f*^−/−^ pups, whereas no death occurred in similarly treated *Tmprss11f*^+/+^ pups in 8 h (Fig. 8B). In histological analysis, we did not detect morphological differences in skin sections between *Tmprss11f*^+/+^ and *Tmprss11f*^−/−^ pups (Fig. 8C). Stratum corneum thicknesses in the sections from *Tmprss11f*^+/+^ and *Tmprss11f*^−/−^ pups were 48.6 ± 9.8 μm (n = 7) and 49.4 ± 7.9 μm (n = 8), respectively (*P* = 0.876). In Western analysis, major cornified envelop proteins, including loricrin, filagrrin, and keratins 1, 5, 10 and 14, from *Tmprss11f*^+/+^ and *Tmprss11f*^−/−^ mice were similar in expression levels and molecular sizes (Fig. 8D). In metabolic studies, water-drinking in adult *Tmprss11f*^−/−^ mice was more frequent than that in *Tmprss11f*^+/+^ controls (166.6 ± 37.9 *vs*. 124.4 ± 33.0 times in 5 days; *P* = 0.033) (Fig. 8E). The total water intake by *Tmprss11f*^−/−^ mice was more than that by *Tmprss11f*^+/+^ mice (18.6 ± 6.0 *vs*. 15.4 ± 5.5 mL in 5 days). The difference, however, did not reach statistical significance (*P* = 0.282) (Fig. 8F). No significant differences in food intake, oxygen consumption and energy expenditure were found between *Tmprss11f*^+/+^ and *Tmprss11f*^−/−^ mice (Supplemental Table 1). These results indicate that HAT-L4 is important in epidermal barrier function to prevent body fluid loss, especially in newborn mice.

## Discussion

TTSPs are distinct members in the trypsin superfamily^4^,^5^. Increasing evidence supports the idea that TTSPs play key roles in physiological homeostasis^6^,^10^. HAT-L4 is a TTSP identified by genomic sequence analysis^4^. To date, its biochemical property, cellular distribution and physiological function remain unknown. In this study, we expressed and analyzed human HAT-L4 in CHO cells. In SDS-PAGE and Western blotting, recombinant HAT-L4 migrated predominantly as a band of ~48 kDa, consistent with the calculated mass of human HAT-L4 protein that lacks predicted *N*-glycosylation sites. The predominant single protein band on Western blots under reducing conditions indicates that HAT-L4 expressed in the CHO cells remained primarily as a one-chain zymogen. By flow cytometry, recombinant HAT-L4 protein was found on the surface of the transfected CHO cells, confirming the expected cell membrane topology of HAT-L4 as a type II transmembrane protein.

To examine HAT-L4 tissue distribution, we surveyed 32 mouse tissues by RT-PCR. HAT-L4 mRNA was detected in multiple tissues, including skin, eye, trachea, tongue, esophagus, bladder, testis, uterus and vagina. Our results are in agreement with findings of a previous study, in which HAT-L4 mRNA was identified by RT-PCR in mouse tissues such as tongue, testis, skin, and bladder^34^. By IHC, we found HAT-L4 protein expression in epithelial cells and exocrine glands in human tissues such as skin, esophagus, trachea, testis and placenta. In skin sections, HAT-L4 staining was positive in the stratum corneum, epidermal keratinocytes, sebaceous glands and sweat glands. To date, several TTSPs, including matriptase, HAT, TMPRSS11A, TMPRSS13 and corin, have been found in skin tissues, mostly in squamous epithelium and/or hair follicles^34^,^36^,^37^,^38^. The abundant HAT-L4 expression in sebaceous and sweat glands appears distinct among the human TTSP family members.

To determine the physiological importance of HAT-L4 *in vivo*, we generated HAT-L4-deficient mice by disrupting the *Tmprss11f* gene. The intended gene targeting was verified by PCR genotyping, DNA sequencing, and RT-PCR analysis of HAT-L4 mRNA expression. Although HAT-L4 is expressed in the testis and uterus, HAT-L4-deficient male and female mice were fertile and had similar litter sizes to that of WT mice. HAT-L4-deficient mice also had a seemingly normal lifespan. In blood chemistry analysis, all parameters tested were normal in *Tmprss11f*^−/−^ mice. In necropsy and histological analysis, no abnormalities were identified in major organs from these mice. Particularly, in organs where HAT-L4 expression was detected, including skin, trachea, tongue, esophagus, testis and eye, tissue morphology was indistinguishable between *Tmprss11f*^+/+^ and *Tmprss11f*^−/−^ mice. These data indicate that HAT-L4 is not essential for embryonic development, post-natal growth and long-term survival in mice.

Extracellular proteolysis mediated by membrane-bound serine proteases is critical for epidermal differentiation and maturation^12^,^35^,^39^,^40^. In mice, deficiency in matriptase, prostasin and TMPRSS13 impairs stratum corneum formation and epidermal barrier function^38^,^39^,^41^,^42^,^43^,^44^,^45^. Mutations in the *ST14* gene encoding matriptase have been identified in patients with a skin disorder, called autosomal recessive ichthyosis with hypotrichosis^18^,^19^,^46^,^47^. In our study, we found no obvious differences between *Tmprss11f*^+/+^ and *Tmprss11f*^−/−^ mice in hair color and skin histology. There were no apparent defects in hair growth and wound healing in the KO mice. Similar results of hair drying and body temperature recovery were observed in adult *Tmprss11f*^+/+^ and *Tmprss11f*^−/−^ mice in a water bath test, indicating that HAT-L4 is not critical for the skin function to repel external water and regulate body temperature in adult mice.

By IHC, we found abundant HAT-L4 expression in sebaceous glands. The primary function of sebaceous glands is to produce a surface lipid layer consisting of triglycerides, wax esters, squalene, free fatty acids, and cholesterol^48^,^49^. Such a lipid layer is essential for preventing trans-epidermal body fluid loss. In histological analysis, we did not find significant differences between *Tmprss11f*^+/+^ and *Tmprss11f*^−/−^ mice in sebaceous gland morphology and number. In a trans-epidermal fluid loss test performed at 37 °C, greater body fluid loss and mortality were found in *Tmprss11f*^−/−^ newborn pups that had no access to milk-drinking. Previously, excessive body fluid loss was reported in matriptase-, prostasin- and TMPRSS13-deficient newborn mice that had impaired stratum corneum formation^19^,^38^,^43^,^45^. In our study, the epidermal structure was similar in *Tmprss11f*^+/+^ and *Tmprss11f*^−/−^ newborn mice. Western analysis found similar expression levels of major cornified envelop proteins, including loricrin, filaggrin and keratins 1, 5, 10 and 14 in these mice. These data indicate that HAT-L4 deficiency did not significantly alter the epidermal structure. Given the abundant HAT-L4 expression in sebaceous glands, it is possible that HAT-L4 deficiency may impair sebaceous gland function, thereby compromising the epidermal barrier function to prevent body fluid loss in newborn mice in a high-temperature environment. Notably, we found no excessive loss of *Tmprss11f*^−/−^ pups between the birth and weaning, indicating that the role of HAT-L4 in the epidermal barrier function is less critical in a room-temperature environment where milk-drinking is readily accessible. We also noticed in our metabolic studies that adult *Tmprss11f*^−/−^ mice drank water more frequently than *Tmprss11f*^+/+^ mice did. Conceivably, more frequent water drinking may compensate the extra trans-epidermal body fluid loss caused by HAT-L4 deficiency.

In summary, our study reveals a role of HAT-L4 in the epidermal barrier function to prevent body fluid loss, providing another example of cell membrane-bound serine proteases in epidermal development and function. Currently, the molecular basis underlying the role of HAT-L4 in the epidermal barrier function remains unknown. The zymogen activation mechanism and substrate specificity of HAT-L4 have not been determined. In principle, HAT-L4 deficiency could impair sebaceous gland excretion and/or lipid composition, which may compromise the epidermal barrier function but are difficult to be detected by the methods used in this study. Our findings should encourage further investigations to understand how HAT-L4 participates in the epidermal barrier function. Such studies may provide new insights into protease-mediated pathways in skin biology and disease.

## Methods

### Anti-HAT-L4 Antibodies

A cDNA fragment encoding human HAT-L4 protease domain (amino acids 196–433 based on NCBI accession number NP_997290.2) was amplified from the human leukemia cell line MEG-01 by RT-PCR. The PCR fragment was sequenced and inserted into pQE30 expression vector encoding a C-terminal His tag (Qiagen). Recombinant HAT-L4 protein was expressed in *Escherichia coli*, purified with a Ni-NTA agarose column (GE Healthcare Life Sciences), and verified by SDS-PAGE and Coomassie blue staining. The purified HAT-L4 protein was used to immunize BALB/c mice to make monoclonal antibodies. Positive hybridoma cells were identified by ELISA using the purified recombinant HAT-L4 protein and used to produce ascites in mice. Monoclonal antibodies were purified from the ascites using a protein-G-Sepharose 4B column (GE Healthcare Life Sciences) and analyzed by Western blotting, immunostaining and flow cytometry. In Western blotting and immunohistochemical analyses, the antibodies recognized human HAT-L4 protein but not mouse HAT-L4 protein.

### Transfection, Western Blotting, and Flow Cytometry

A cDNA encoding the full-length human HAT-L4 amplified from MEG-01 cells was inserted into pcDNA expression plasmid that encodes a C-terminal V5 tag. The resultant plasmid pcDNA-HAT-L4 and control pcDNA vector were transfected into CHO cells with FuGENE reagents (Roche Diagnostics). After 24 h at 37 °C, the cells were lysed in a solution containing 50 mmol/L Tris-HCl, pH 8.0, 150 mmol/L NaCl, 1% (v/v) Triton X-100 and a protease inhibitor cocktail (1:100 dilution, Sigma)^50^. HAT-L4 protein was analyzed by SDS-PAGE and Western blotting under reducing conditions using an anti-V5 antibody (Invitrogen) and an anti-HAT-L4 antibody described above. To analyze HAT-L4 protein on the cell surface, the transfected CHO cells were detached with EDTA and incubated with the anti-V5 and anti-HAT-L4 antibodies or mouse IgG control. After washing and incubation with a phycoerythrin-conjugated secondary antibody, the cells were analyzed by flow cytometry (FACSCalibur, BD Biosciences), as described previously^51^.

### Immunohistochemistry

All anonymous human tissues were from a biobank from the Department of Pathology of Soochow University. The study was conducted in accordance with the Soochow University’s ethics committee guidelines. Sections were made from formalin-fixed and paraffin-embedded human tissues, de-paraffinized with xylene, and rehydrated with graded ethanol solutions. The sections were boiled in 10 mM citrate buffer (pH 6. 0) for 2 min for antigen retrieval, treated with 5% (w/v) bovine serum albumin (BSA) in phosphate buffered solution (PBS) at 37 °C for 1 h to block non-specific binding, and then incubated with an anti-HAT-L4 antibody (1:450 dilution) at 4 °C overnight. In negative controls, the primary antibody was replaced by 5% BSA. After washing with PBS, an HRP-conjugated secondary antibody was added. After incubation and washing, an HRP substrate solution containing 3, 3′-diaminobenzidine was added. The sections were examined under a light microscope (Olympus, DP73).

### HAT-L4 mRNA Expression in Mouse Tissues

All animal procedures were carried out in accordance with the guidelines for the ethical treatment and handling of animals in research and approved by the Animal Care and Use Committees at the Soochow University. In RT-PCR experiments, tissues from wild-type (WT) mice (C57BL/6; 10 males and 6 females; 2 months old) were used to isolate total RNAs using the Trizol reagent (Invitrogen). The RNAs were used as templates to make cDNAs using the RevertAid First Strand cDNA Synthesis kit (Fermentas). PCR amplification of *Tmprss11f* transcripts was performed using forward (5′-ACC TAA AAC AAG TGT GTT CG-3′) and reverse (5′-TCG ATA CTT AGT TAC TCT GG-3′) primers in a protocol of 35 cycles with 1 min denaturation at 95 °C, 30 s annealing at 55 °C, and 30 s elongation at 72 °C. PCR products were analyzed by agarose gel electrophoresis.

### Generation of *Tmprss11f* KO Mice

Mice with a disrupted *Tmprss11f* allele were generated by CRISPR/Cas9-based techniques at the Model Animal Research Center of Nanjing University, China^52^. Briefly, a guide RNA (gRNA) sequence targeting exon 4 of the mouse *Tmprss11f* gene was ligated to pUC57-T7-gRNA vector and transcribed by the MEGAshortscript kit (Ambion). The pST1374-Cas9-N-NLS-flag-linker vector was linearized by Age1 enzyme and *in vitro* transcribed using the T7 Ultra kit (Ambion). A mixture of the Cas9 mRNA and gRNA was injected into mouse zygotes that were cultured in Hamster Embryo Culture Medium-10 with 10% fetal bovine serum (Hyclone) at 37 °C in an incubator with 5% CO_2_. The cleaved embryos at two-cell to blastocyst stages were transferred into recipient mice. The offspring was genotyped by PCR with forward (5′-GTC ATG TAA ACC AAG TGT GTG ATG-3′) and reverse (5′-GAG ATA TAG ACT GCC AGC ATA GC-3′) primers. The PCR products were verified by DNA sequencing. Positive mice with the disrupted *Tmprss11f* gene were identified and bred with C57BL/6 mice. Heterozygous offspring was identified by PCR genotyping with the primers TMPRSS11fT85F (5′-CAA GTC ATT TTA TTA CCT CGC CTC T-3′) and TMPRSS11fT85R (5′-AAA CTC CCT TGA TGA CCT GAT TCC A-3′). The heterozygous mice were bred to produce *Tmprss11f* null mice and littermates for further analysis.

### RT-PCR Analysis of HAT-L4 mRNA in KO Mice

Tongue tissues were collected from adult male *Tmprss11f*^*+*/*+*^ and *Tmprss11f*^−/−^ mice and homogenized in Trizol reagent (Invitrogen). Total RNAs were isolated and used for RT-PCR using the forward (5′-GCC CTC GGA TTT GGT AGG C-3′) and reverse (5′-CCG TGT GTG TGA CCT TGT CT-3′) primers in 35 cycles of 1 min denaturation at 95 °C, 30 s annealing at 55 °C, and 1 min elongation at 72 °C. PCR products were analyzed by agarose gel electrophoresis.

### Blood Chemistry Analysis

Blood samples were collected from 2–4 month-old male and female *Tmprss11f*^*+*/*+*^ and *Tmprss11f*^−/−^ mice. Blood chemistry analysis was done at the Clinic Laboratory of the Second Affiliated Hospital of Soochow University. Blood cell counts were examined using a hematology analyzer (Siemens, ADVIA 2120i).

### Histological Analysis of Mouse Tissues

Tissues from 2–4 month-old *Tmprss11f*^*+*/*+*^ and *Tmprss11f*^−/−^ mice were dissected, fixed with formalin, and embedded in paraffin. Sections (5 μm in thickness) were cut and stained with H&E. Histological examination was done under a light microscope (Olympus, DP73).

### Hair Growth and Wound Healing

A dorsal area was depilated in 3–4 month-old male mice. Hair growth was inspected daily and pictures were taken at days 1, 10 and 20. To study wound healing, an excisional round wound of 1 cm in diameter was created. Wound healing was inspected daily and recorded by a camera (Canon, 500D). Wound areas were analyzed using Image-Pro Plus (Media Cybernetics) and wound healing rates were calculated.

### Water Repulsion Test

A water repulsion test was performed to assess the skin function in water repulsion and thermoregulation, based on a published protocol^53^. Briefly, 3-month-old male mice were placed in a water tank at 30 °C. After 2 min, the mice were taken out and placed on paper towels at room temperature to eliminate excessive water. Body weight and rectal temperature were measured before and after the water bathing. The water remained in hairs was calculated by comparing the body weight before and after the water bathing.

### Trans-epidermal Fluid Loss Test

A trans-epidermal fluid loss test was performed in newborn mice to assess the skin barrier function to prevent excessive body fluid loss, based on a published protocol^37^. Briefly, <12-h-old newborn pups were taken away from their mothers and placed in a 37 °C incubator. Body weights of individual pups were measured hourly over a period of 5 h. The trans-epidermal fluid loss was calculated based on body weight decrease over time.

### Water Intake

Twenty-week-old male mice were placed in a comprehensive laboratory animal monitoring system (Columbus Instruments, Oxymax) and fed a regular chow diet and drinking water *ad libitum*. After the mice were acclimated to the system for >24 h, water intake and drinking frequency were recorded.

### Western Analysis of Skin Proteins

To analyze epidermal proteins in mice, skin tissues from newborn (<12 h old) mice were homogenized in a lysis solution^38^. Protein samples were analyzed by SDS-PAGE and Western blotting, as described previously^51^. Western blots were incubated with primary antibodies against loricrin (Abcam, ab85679), filaggrin (BioLegend, 905804), and keratins 1 (Abcam, ab185628), 5 (Abcam, ab52635), 10 (Abcam, ab76318) and 14 (Abcam, ab181595). A horseradish-peroxidase (HRP)-conjugated antibody (Shunshine Bio, SN134) was used as a secondary antibody.

### Statistical Analysis

The analysis was done using the Prism 6 (GraphPad) and SPSS18 software. All data are presented as mean ± S.D. Comparisons between two groups were done using Student’s *t* test. χ^2^ test was used to examine genotype distribution among offspring from heterozygous mating. The Mantel–Cox test was used to compare long-term survival rates between WT and KO mice. A *P* value of <0.05 was considered to be statistically significant.

## Additional Information

**How to cite this article:** Zhang, Z. *et al*. The Transmembrane Serine Protease HAT-like 4 Is Important for Epidermal Barrier Function to Prevent Body Fluid Loss. *Sci. Rep.* **7**, 45262; doi: 10.1038/srep45262 (2017).

**Publisher's note:** Springer Nature remains neutral with regard to jurisdictional claims in published maps and institutional affiliations.

## Supplementary Material

Supplementary Information
