# Author Correction: The Transmembrane Serine Protease HAT-like 4 Is Important for Epidermal Barrier Function to Prevent Body Fluid Loss

**DOI:** 10.1038/s41598-020-62767-5

**Published:** 2020-04-10

**Authors:** Zhiwei Zhang, Yae Hu, Ruhong Yan, Liang Dong, Yizhi Jiang, Zhichao Zhou, Meng Liu, Tiantian Zhou, Ningzheng Dong, Qingyu Wu

**Affiliations:** 1 Cyrus Tang Hematology Center, MOE Engineering Center of Hematological Disease, Collaborative Innovation Center of Hematology, Soochow University, Suzhou, China; 2 MOH Key Lab of Thrombosis and Hemostasis, Jiangsu Institute of Hematology, the First Affiliated Hospital of Soochow University, Suzhou, China; 3 Molecular Cardiology, Cleveland Clinic, Cleveland, Ohio USA

Correction to: *Scientific Reports* 10.1038/srep45262, published online 24 March 2017

This Article contains an error in Figure 8D, where the wrong image for keratin 1 was inadvertently used. The correct versions of Figure 8D and Supplementary Figure 1, which shows the original Western blots, are shown below as Figures 1 and 2 respectively.

**Figure 1:**
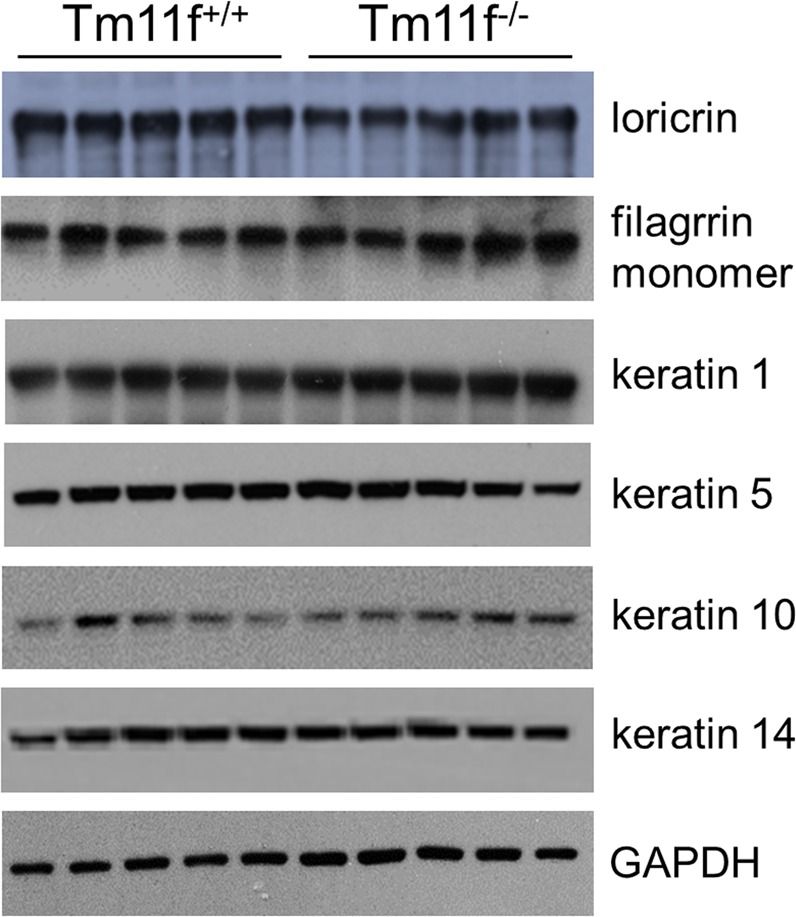
.

**Figure 2:**
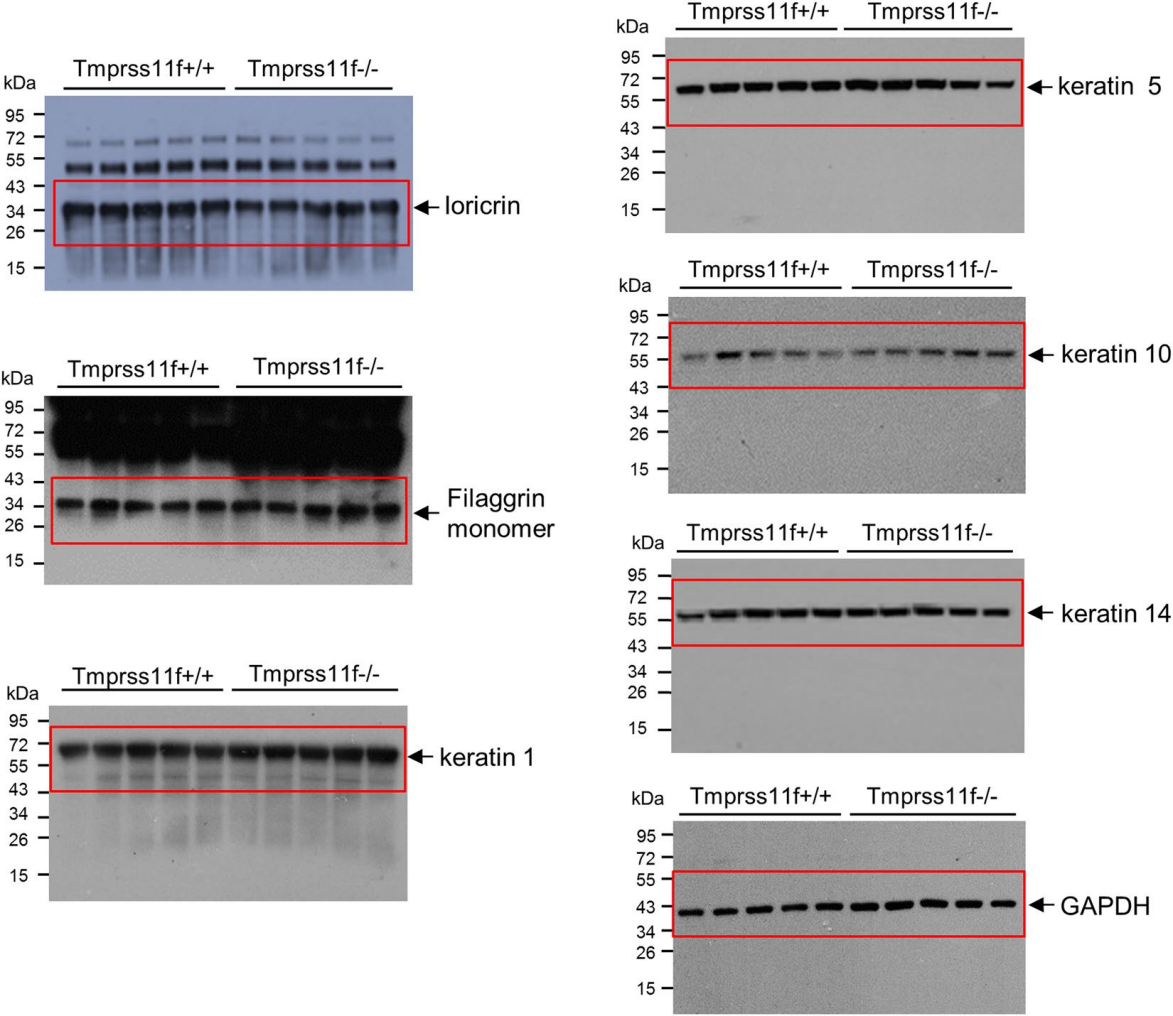
.

