# Supplementary material for: The Transmembrane Serine Protease HAT-like 4 Is Important for Epidermal Barrier Function to Prevent Body Fluid Loss
Source: Sci Rep. 2017 Mar 24;7:45262. doi: 10.1038/srep45262 (PMC5364460; doi:10.1038/srep45262)

Supplementary Information

The Transmembrane Serine Protease HAT-like 4 Is Important for Epidermal Barrier Function to Prevent Body Fluid Loss

Zhiwei Zhang[1], Yae Hu[1], Ruhong Yan[1], Liang Dong[1], Yizhi Jiang[1,2], Zhichao Zhou[1,2], Meng Liu[1], Tiantian Zhou[1], Ningzheng Dong[1,2], and Qingyu Wu[1,3]

Supplementary Table 1.

Food intake, oxygen consumption and energy expenditure in *Tmpress11*$^{-/-}$ mice

		Tmpress11f$^{+/+}$	***Tmress11f***$^{-/-}$
**Food intake**	Total weight (g)	27.1 ± 8.7	28.6 ± 10.5
	Frequency (time)	157.6 ± 21.8	154.5 ± 36.1
**Oxygen consumption (mL/h/kg)**	Light cycle	3703.3 ± 242.3	3570.9 ± 382.1
	Dark cycle	4452.5 ± 173.6	4289.6 ± 309.3
**Energy expenditure (cal/h)**	Light cycle	462.8 ± 45.2	456.5 ± 21.5
	Dark cycle	569.9 ± 41.6	561.3 ± 37.5

Differences between *Tmprss11*$^{+/+}$ and *Tmprss11*$^{-/-}$ mice are not statistically significant by Student's *t* test.

Supplementary Fig. 1. Original Western blots

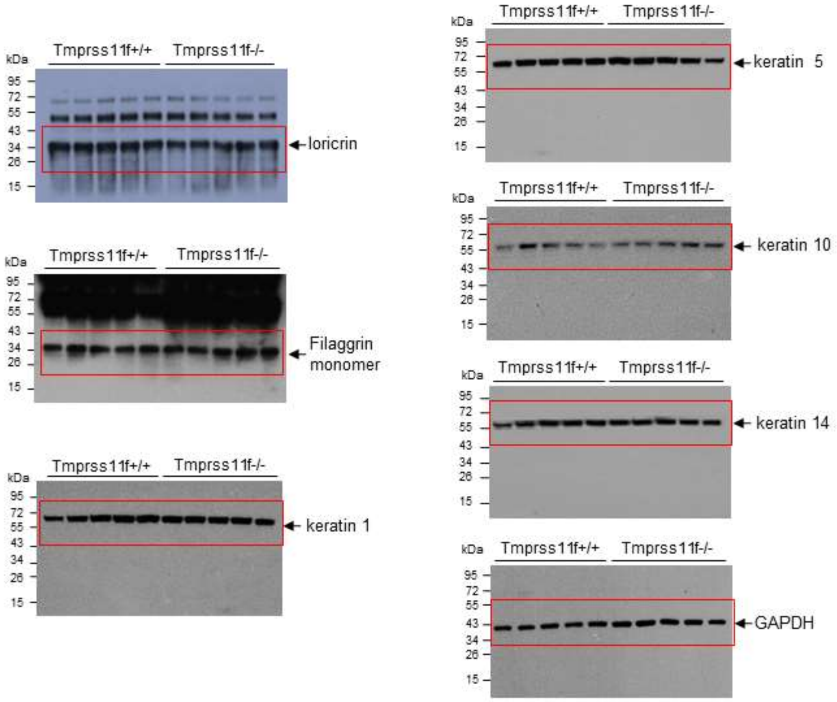

Supplement: Supplementary Information [file srep45262-s1.pdf]
